# Non-invasive adjunctive diagnostic models incorporating high-frequency ultrasound and shear wave elastography for pediatric Duchenne muscular dystrophy

**DOI:** 10.3389/fgene.2026.1912996

**Published:** 2026-10-21

**Authors:** Kun-Kun Qiang, Si-Ran Zhang, Hong Luo, Qing-Yun Song

**Affiliations:** 1 Department of Diagnostic Ultrasound, West China Second University Hospital, Sichuan University, Chengdu, China; 2 Key Laboratory of Obstetric & Gynecologic and Pediatric Diseases and Birth Defects of Ministry of Education, Chengdu, China

**Keywords:** Duchenne muscular dystrophy (DMD), genetic diseases, HFUS-SWE model, high-frequency ultrasound (HFUS), shear wave elastography (SWE)

## Abstract

**Background:**

This study aimed to comprehensively characterize skeletal muscle properties in pediatric patients with Duchenne muscular dystrophy (DMD) using high-frequency ultrasound (HFUS) and shear-wave elastography (SWE), and to construct and compare diagnostic models based on these modalities to evaluate their potential as non-invasive adjunctive tools for assessing muscular involvement.

**Methods:**

A total of 53 genetically or pathologically confirmed pediatric DMD patients and 61 age- and sex-matched healthy controls were enrolled. The bilateral biceps brachii and quadriceps femoris muscles were examined using HFUS and SWE. Inter-observer reliability was assessed using the intraclass correlation coefficient (ICC). Principal component analysis (PCA) was applied to HFUS parameters for dimensionality reduction, followed by logistic regression to establish a standardized HFUS diagnostic model. A separate SWE model was constructed using univariate and multivariate logistic regression. Receiver operating characteristic (ROC) curve analysis and decision curve analysis (DCA) were used to evaluate and compare the diagnostic performance of the individual and combined models.

**Results:**

The HFUS model achieved an area under the curve (AUC) of 0.875 (95% CI: 0.807–0.943), with 88.9% sensitivity and 81.9% specificity. The SWE model showed an AUC of 0.846 (95% CI: 0.763–0.909), with 69.8% sensitivity and 75.4% specificity, with no significant difference between the two (*P* = 0.494). Notably, the combined HFUS-SWE model yielded superior diagnostic accuracy (AUC = 0.947, 95% CI: 0.885–0.981; sensitivity = 82.2%, specificity = 95.1%), significantly outperforming each individual model (both *P* < 0.001). DCA demonstrated that the combined model provided a greater net benefit across all threshold probabilities.

**Conclusion:**

Both the HFUS and SWE models demonstrated high diagnostic accuracy for DMD. Their combination significantly enhances diagnostic efficacy, offering a promising non-invasive adjunctive strategy for evaluating muscular involvement in children with DMD.

## Introduction

Duchenne muscular dystrophy (DMD) is one of the most common fatal neuromuscular diseases, inherited in an X-linked recessive pattern. Its pathogenesis involves deletions, duplications, or point mutations in the dystrophin gene (McCourt et al.,2023). Dystrophin is a core component of the muscle fiber cytoskeleton; its functional absence leads to repeated cycles of muscle fiber necrosis and regeneration, which eventually fail, resulting in replacement by fibrotic tissue and fat infiltration, accompanied by cardiomyopathy and respiratory muscle involvement (Birnkrant et al., 2018; Birnkrant et al., 2018a). The global incidence of DMD is approximately one in 3,500–5,000 male live births (Birnkrant et al., 2018). In advanced stages, patients develop progressive degenerative changes and contractures, particularly affecting the paraspinal and other axial muscles, which often lead to severe scoliosis and significant functional decline (Muntoni et al., 2023). Given its prolonged course and poor prognosis, DMD imposes a substantial burden on affected families and severely impacts children’s physical and mental health. Therefore, non-invasive adjunctive diagnostic and therapeutic strategies are crucial for extending survival and improving quality of life (Landfeldt et al., 2019).

Currently, DMD diagnosis relies on muscle biopsy, genetic testing, clinical symptom assessment, and serum muscle enzyme assays. Muscle biopsy and genetic testing are regarded as the diagnostic “gold standards”. However, muscle biopsy in children is technically challenging and has low parental acceptance (Vandekerckhove et al., 2020; Thavorntanaburt et al., 2018; Schiava et al., 2025). While genetic testing remains the definitive standard, particularly for guiding targeted therapies, it is resource-intensive and may not be readily accessible as a first-line screening tool in all clinical settings. Thus, there is an urgent need for a cost-effective, non-invasive, and widely accessible triage tool to accurately identify children at high risk for DMD, thereby optimizing the use of confirmatory genetic testing (Nallamilli et al., 2021).

DMD is characterized by a specific, predictable pattern of muscle involvement. Pathologically, muscle degeneration and fibrofatty replacement are known to occur earlier and more severely in the proximal lower limb muscles (particularly the quadriceps and gluteal groups) compared to the distal upper limb muscles, a pattern consistently demonstrated in MRI and histological studies. The biceps brachii, representing an upper limb muscle, and the quadriceps femoris, representing a lower limb muscle, are commonly affected and are easily accessible for ultrasound examination. Furthermore, their superficial location ensures high image quality and reproducibility with HFUS and SWE. These muscles are also frequently selected in pediatric neuromuscular ultrasound studies, allowing for comparability with existing literature.

Ultrasound technology is increasingly used in the diagnosis of neuromuscular diseases due to its ease of operation, high sensitivity, and non-invasiveness (Wijntjes and van Alfen, 2021). However, research specifically targeting DMD with ultrasound remains limited, and the establishment of relevant diagnostic models is urgently needed. This study aims to develop a non-invasive adjunctive diagnosis approach for DMD through innovative imaging methods.

HFUS and SWE offer distinct and complementary advantages in muscle disease diagnosis. HFUS excels in quantifying fat infiltration in muscular dystrophy with high accuracy and demonstrates excellent diagnostic performance in inflammatory myopathies (Vicino et al., 2024). Numerous studies have employed HFUS to measure key morphological parameters such as muscle cross-sectional area and thickness, which show the strongest correlation with muscle strength (Van den Broeck et al., 2021; Gupta et al., 2022; Liegnell et al., 2021). SWE, a user-friendly quantitative technique with high reproducibility, assesses muscle stiffness by measuring shear wave velocity (SWV) and has excellent applicability in diagnosing pediatric muscular disorders (Goo et al., 2020; Bastijns et al., 2020; Cebula et al., 2021; Miller et al., 2021). In this investigation, longitudinal SWE measurements were obtained from various muscle groups. Logistic regression was then used to construct robust diagnostic models, and their efficacy was evaluated for DMD. Given their ability to accurately assess changes in muscle morphology and function, HFUS and SWE have become prominent research foci in muscle disease diagnosis. This study aimed to develop an economical and effective non-invasive adjunctive diagnostic method for DMD, providing new insights for early clinical intervention, disease course monitoring, and ultimately improving prognosis.

## Materials and methods

### Study population

This retrospective study enrolled DMD patients who visited the Department of Pediatric Neurology and Rehabilitation at West China Second University Hospital between January 2019 and September 2024 as the case group. DMD diagnosis was confirmed by genetic testing and/or muscle biopsy.

### Exclusion criteria

(1) Children with intellectual disabilities who are unable to cooperate to complete the examination; (2) Children with a history of using hormonal drugs for treatment currently or within the past 6 months; (3) Children who have received physical training for 1 month or more within the past 3 months and those who have undergone surgical orthopedic operations within the past half year; (4) Children suffering from various diseases that obviously affect their physical fitness; (5) Children without the informed consent of their guardians.

Age-matched healthy boys with normal physical examinations and no neuromuscular or other diseases were enrolled as the control group. This study was approved by the Ethics Review Committee of West China Second Hospital of Sichuan University (Approval No. 2024k254), and the informed consents were obtained from the guardians. The study was conducted in accordance with the Declaration of Helsinki.

### Instruments and methods

Basic information including weight, body mass index (BMI), sex, age, and height were collected for all participants. Clinical manifestations (walking ability, muscle strength, family history, maternal carrier status) and disease progression over the past 3 months were also recorded.

Ultrasound examinations were performed using a Mindray Resona 7 system equipped with SWE and a high-frequency linear array probe (5–14 MHz). All children first underwent HFUS, followed by SWE, in a naturally relaxed state. The bilateral biceps brachii and quadriceps femoris were examined. For HFUS, transverse and longitudinal scans were performed along the muscle plane to observe internal echogenicity, texture, blood flow signals, and space-occupying lesions, followed by measurement of morphological parameters (muscle thickness and cross-sectional area). The cross-sectional area was measured to include as much muscle fiber tissue as possible, representing the sum of the cross-sectional areas of all muscle fibers within the detected muscle (Figures 1, 2).

**FIGURE 1:**
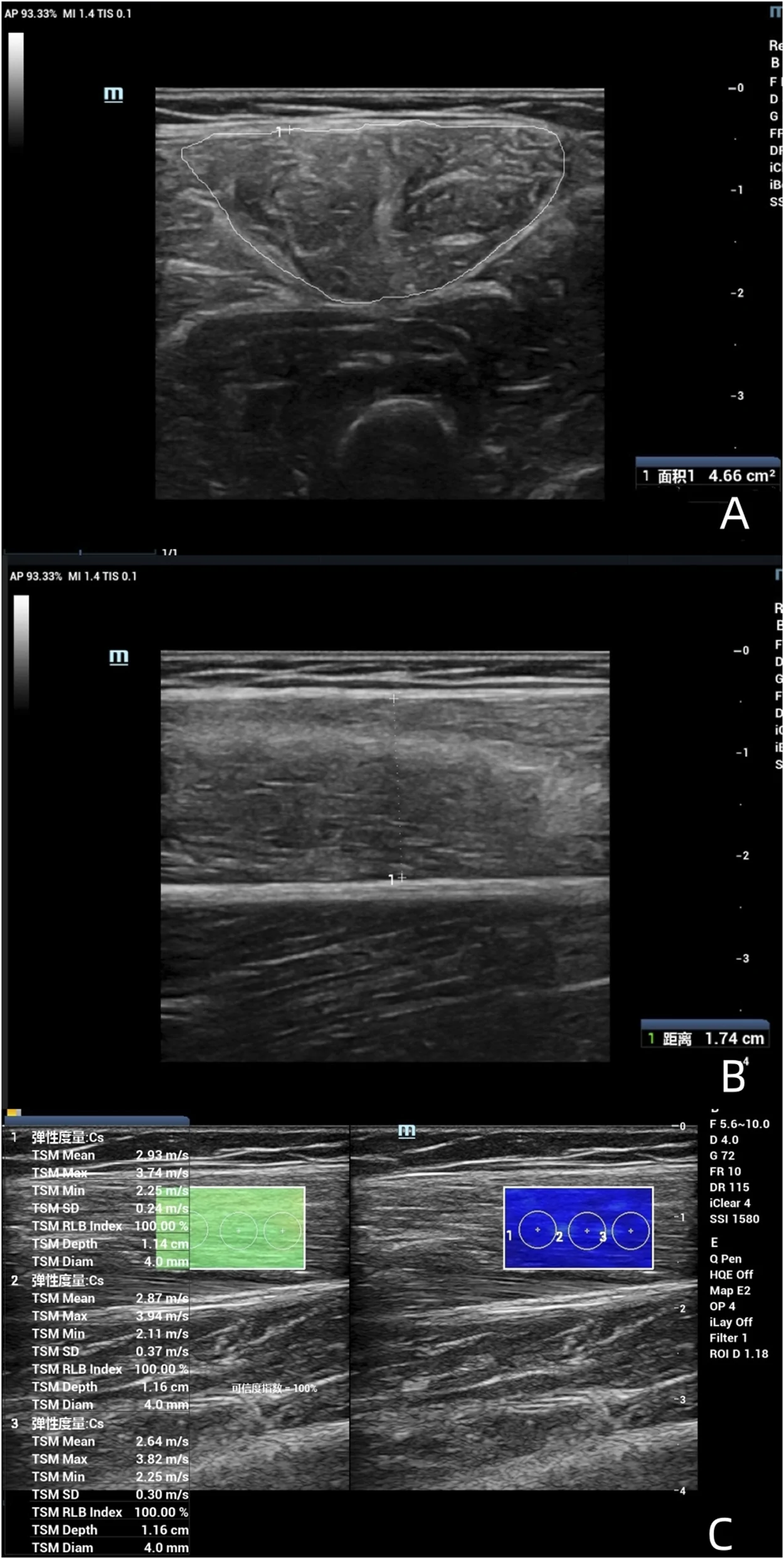
A 5-year-old boy patient underwent high - frequency ultrasound (HFUS) and shear - wave elastography (SWE). **(A)** The transverse cross-sectional area of the right rectus femoris muscle. **(B)** The thickness of the right rectus femoris muscle. **(C)** The mean shear wave velocity of the right rectus femoris muscle.

**FIGURE 2:**
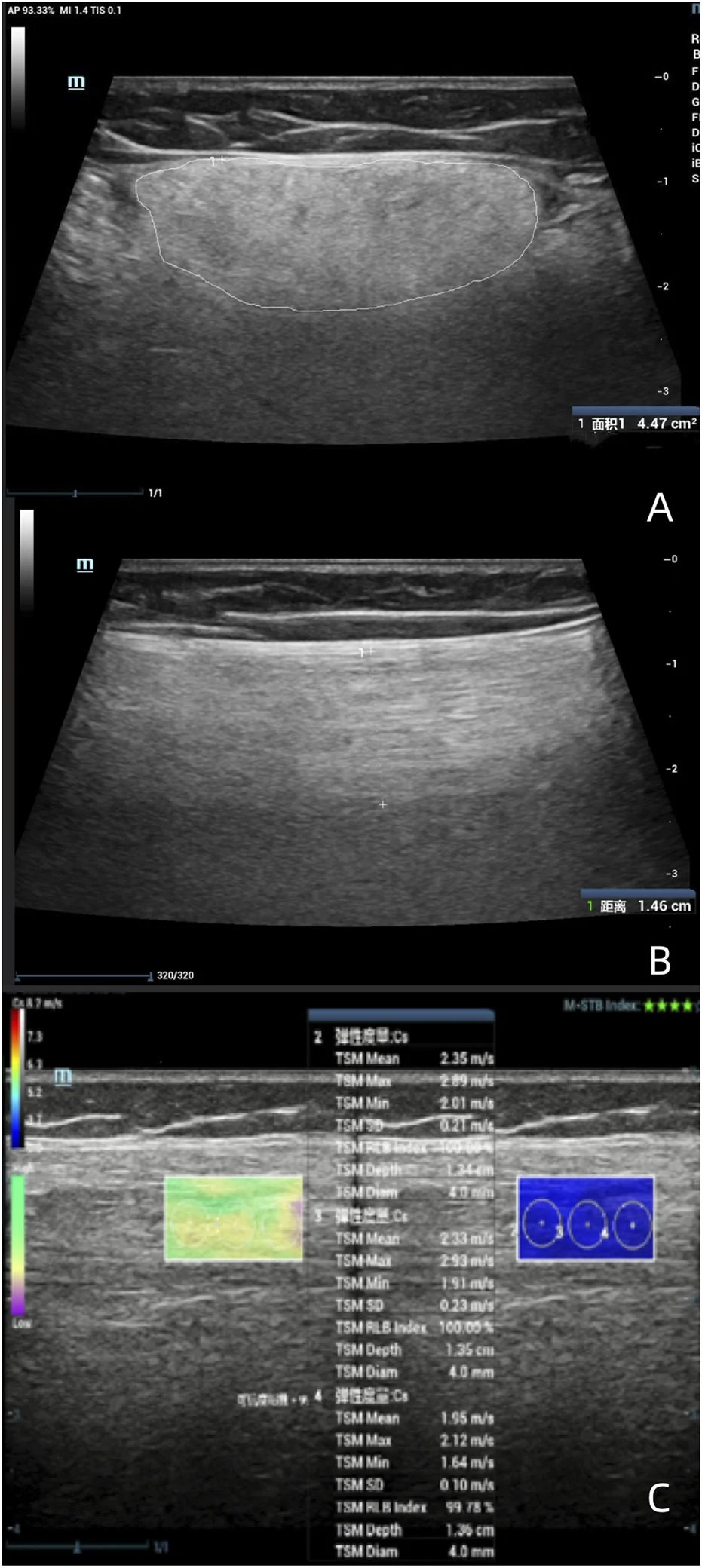
A 10-year-old boy patient underwent high-frequency ultrasound (HFUS) and shear-wave elastography (SWE). **(A)** The transverse cross-sectional area of the right rectus femoris muscle. **(B)** The thickness of the right rectus femoris muscle. **(C)** The mean shear wave velocity of the right rectus femoris muscle.

All examinations were performed by a single certified sonographer with over 5 years of experience in pediatric neuromuscular imaging, using a standardized protocol. Patients were positioned supine with their arms in neutral rotation and legs fully extended and relaxed. For the biceps brachii, the probe was placed at the midpoint between the acromion and the antecubital fossa. For the quadriceps femoris, measurements were taken at the midpoint between the anterior superior iliac spine and the superior border of the patella. Anatomical muscle borders were identified in the transverse plane to ensure the cross-sectional area included the entire muscle belly, excluding surrounding fascia.

All acquired images were independently analyzed and measured independently by two pediatric radiologists with over 10 years of experience, who were blinded to the participants’ clinical status and group allocation. The final value for each parameter was the average of the measurements obtained by the two readers. The inter-observer reliability was assessed using the intraclass correlation coefficient (ICC) (Table 1).

**TABLE 1:** Consistency analysis between the same physician and different physicians.

Group	Muscle type	Parameter	The same physician ICC		Different physicians ICC	
			Left	Right	Left	Right
Case group	Biceps brachii	Csmean	0.804	0.865	0.967	0.661
	​	Thickness	0.950	0.972	0.971	0.975
	​	Cross-sectional	0.970	0.944	0.969	0.963
	Rectus femoris	Cmean	0.946	0.910	0.914	0.770
	​	Thickness	0.928	0.978	0.943	0.954
	​	Cross-sectional	0.991	0.987	0.987	0.979
	Musculus medius femoris	Cmean	0.753	0.827	0.809	0.586
		Thickness	0.982	0.981	0.980	0.975
		Cross-sectional	0.983	0.947	0.982	0.983
	Musculus medialis femoris	Csemans	0.825	0.873	0.657	0.885
		Thickness	0.973	0.980	0.957	0.968
		Cross-sectional area	0.977	0.972	0.970	0.969
	Lateral femoral muscle	Csmean	0.859	0.847	0.870	0.857
		Thickness	0.968	0.933	0.962	0.939
		Cross-sectional	0.971	0.946	0.960	0.932
Control group	Biceps brachii	Csmean	0.804	0.824	0.879	0.801
	​	Thickness	0.968	0.986	0.964	0.976
	​	Cross-sectional	0.968	0.983	0.967	0.973
	Rectus femoris	Cmean	0.874	0.729	0.800	0.772
	​	Thickness	0.985	0.991	0.984	0.997
	​	Cross-sectional	0.988	0.993	0.988	0.990
	Musculus medius femoris	CSM	0.596	0.564	0.499	0.531
		Thickness	0.994	0.996	0.993	0.992
		Cross-sectional	0.994	0.990	0.988	0.990
	Musculus medialis femoris	Csemans	0.651	0.857	0.605	0.627
		Thickness	0.996	0.995	0.995	0.998
		Cross-sectional	0.988	0.993	0.990	0.983
	Lateral femoral muscle	Csmean	0.723	0.839	0.726	0.833
		Thickness	0.982	0.992	0.970	0.977
		Cross-sectional	0.988	0.990	0.983	0.987

Mean shear wave velocity (Csmean) is expressed in meters per second (m/s). High-frequency parameters Muscle thickness is measured in centimeters (cm). Cross-sectional area is calculated in square centimeters (cm^2^).

### Statistical analysis

Data processing was performed using Stata 14.0 and SPSS 22.0. A *P*-value <0.05 was considered statistically significant. The Kolmogorov-Smirnov test was used to assess normality. Non-normally distributed data were presented as median (interquartile range) and analyzed using the rank-sum test; normally distributed data were presented as mean ± standard deviation and analyzed using the *t*-test. Categorical data were compared using the chi-square test.

To build the HFUS model, we first confirmed significant correlation between variables (KMO value >0.8). PCA was then used for dimensionality reduction. The number of principal components was determined based on eigenvalues (1) and cumulative contribution rates. Logistic regression was performed using these factors to construct the HFUS diagnostic model.

For the SWE model, potential predictors of DMD were screened by univariate logistic regression, followed by multivariate logistic regression to adjust for confounders. Cut-off values for significant variables were determined using the Youden index and were dichotomized to establish the elastographic diagnostic model.

Finally, we calculated the specificity and sensitivity of the two models through the Receiver Operating Characteristic (ROC) curve and the goodness-of-fit through the Hosmer-Lemeshow test. And we compared the efficacy of the two models through the Decision Curve Analysis (DCA) and the comparison of ROC curves.

## Results

### Baseline characteristics

There were no statistically significant differences in weight, BMI, or age between the case and control groups (*P* > 0.05). However, height was significantly different between the two groups (*P* < 0.05) (Table 2).

**TABLE 2:** General clinical data of the case group and the control group.

Varible	Total	Case group	Control group	*P*
Age, year	​	5.96 ± 2.14	6.25 ± 1.80	0.444
​	3–7	36 (67.9%)	47 (77%)	​
​	8–12	17 (32.1%)	14 (23%)	​
Height, cm	​	111.98 ± 12.59	117.87 ± 12.53	0.014^*^
Weight, kg	​	20.84 ± 7.00	22.38 ± 5.30	0.186
BMI (kg/m^2)^	​	16.19 ± 2.40	16.28 ± 4.43	0.903
ALT (U/L)	​	392 (199∼496)	​	​
AST (U/L)	​	268 (177∼415)	​	​
CK (U/L)	​	17,342 (12,506∼19,951)	​	​

^*^ *P* < 0.05, and the difference is statistically significant; BMI: Body Mass Index. ALT: Alanine Transaminase. AST: Aspartate Transaminase. CK: serum creatine kinase.

### Comparison of HFUS and SWE between DMD and control group

When comparing the high-frequency indices between the case group and the control group, there were statistically significant differences in the cross-sectional area of the right biceps brachii, the muscle thickness (MT) and cross-sectional area of the right vastus intermedius, the MT and cross-sectional area of the left vastus intermedius, and the MT of the left vastus medialis. When comparing the elastic indices between the DMD group and the control group, there were statistically significant differences in the Csmean of the right biceps brachii, the Csmean of the right vastus lateralis, the Csmean of the left vastus medialis, and the Csmean of the left vastus intermedius. When comparing the elastic indices between the DMD and control groups, a statistically significant difference was found for the SWV of the right biceps brachii, the right vastus lateralis, the left vastus medialis, and the left vastus intermedius. Critically, for all muscles where a significant difference was observed, the SWV was lower in the DMD group compared to the control group.ol group.ol groupp, indicating reduced muscle stiffness. Except for the thickness of the left and right vastus lateralis and the thickness and cross-sectional area of the left biceps brachii, the muscle thickness and cross-sectional area of the children with DMD were lower than those of the children in the normal group (Table 3).

**TABLE 3:** Comparison of high-frequency and elastic parameters of muscles.

Group	Muscle type	Ultrasound	Parameter	Limb		P
​	​	​	​	Left	Right	​
Case group	Biceps brachii	Elastic index	Csmean	3.65 ± 0.87	3.0 ± 0.55	<0.001**
	​	High-frequency index	Thickness	1.31 ± 0.19	1.27 ± 0.18	0.222
	​	​	Cross-sectional	5.6 ± 1.47	5.04 ± 1.22	0.034*
	Rectus femoris	Elastic index	Csmean	2.56 ± 0.54	2.72 ± 0.57	0.136
	​	High-frequency index	Thickness	1.15 ± 0.09	1.2 ± 0.22	0.271
	​	​	Cross-sectional area	2.97 ± 0.94	3.2 ± 1.06	0.225
	Musculus middle femoris	Elastic index	Csmean	2.83 ± 0.55	2.64 ± 0.43	0.050*
		High-frequency index	Thickness	0.88 ± 0.24	0.9 ± 0.27	0.616
		​	Cross-sectional area	5.41 ± 2.12	5.2 ± 1.96	0.611
	Musculus medialis femoris	Elastic index	Csmean	2.19 ± 0.33	2.13 ± 0.33	0.327
		High-frequency index	Thickness	1.92 ± 0.37	2.09 ± 0.89	0.200
		​	Cross-sectional area	6.01 ± 1.69	5.69 ± 1.76	0.345
	Lateral femoral muscle	Elastic index	Csmean	2.76 ± 0.6	3.06 ± 0.58	0.011*
		High-frequency index	Thickness	1.39 ± 0.21	1.37 ± 0.28	0.746
		​	Cross-sectional	7.63 ± 1.75	7.36 ± 2.13	0.468
Control group	Biceps brachii	Elastic index	Csmean	3.66 ± 0.54	3.47 ± 0.49	0.047*
	​	High-frequency index	Thickness	1.28 ± 0.18	1.24 ± 0.19	0.240
	​	​	Cross-sectional	4.49 ± 0.16	4.42 ± 1.10	0.710
	Rectus femoris	Elastic index	Csmean	2.62 ± 0.46	2.6 ± 0.57	0.790
		High-frequency index	Thickness	1.15 ± 0.2	1.14 ± 0.21	0.828
		​	Cross-sectional	3.0 ± 0.98	3.05 ± 1.06	0.778
	Musculus medialis femoris	Elastic index	Csmean	2.54 ± 0.45	2.64 ± 0.49	0.249
	​	High-frequency index	Thickness	1.15 ± 0.27	1.2 ± 0.29	0.382
	​	​	Cross-sectional	6.53 ± 1.91	6.65 ± 1.97	0.741
	Musculus medialis femoris	Elastic index	Csmean	2.49 ± 0.53	2.24 ± 0.40	0.004*
		High-frequency index	Thickness	2.29 ± 0.55	2.19 ± 0.48	0.274
		​	Cross-sectional	8.08 ± 2.36	5.77 ± 1.79	0.447
	Lateral femoral muscle	Elastic index	Csmean	2.6 ± 0.39	2.66 ± 0.51	0.482
		High-frequency index	Thickness	1.34 ± 0.22	1.33 ± 0.25	0.711
		​	Cross-sectional	8.22 ± 1.87	8.03 ± 2.02	0.587

* *P* < 0.05, statistically significant difference; **P < 0.001, highly statistically significant difference.

### Diagnostic accuracy of the HFUS model

Since the morphological parameters (muscle thickness and cross-sectional area) had a very high relevance, we performed the principal component analysis to reduce the data dimensions. We chose the first four principal components with eigenvalues >1 because these can explain the variation of data more than the individual variables. Then, the first four extracted principal components were analyzed in the univariate and multivariate logistic regression analysis. In multivariate logistic regression analysis, Factor 1 (OR = 2.134, *P* = 0.007) and Factor 2 (OR = 0.132, *P* < 0.001) were selected to enter the model (Table 4).

**TABLE 4:** Single-factor and multi-factor regression analysis for newly generated variables.

Factor	Univariate logistic regression analysis			Multivariate logistic regression analysis		
​	OR	95%CI	P	OR	95%CI	P
Factor 1	1.545	1.026–2.325	0.037	2.134	1.228–3.709	0.007^*^
Factor 2	0.161	0.078–0.331	<0.001	0.132	0.059–0.295	0.001^**^
Factor 3	1.193	0.809–1.759	0.374	​	​	​
Factor 4	0.415	0.154–1.116	0.082	0.351	0.092–1.340	0.126

The HFUS model achieved an AUC of 0.875 (95% CI: 0.807–0.943, *P* < 0.001), with a sensitivity of 88.9% and specificity of 81.9%, indicating good discriminative ability (Figure 3A). The Hosmer-Lemeshow test confirmed good calibration (Figure 3B).

*P < 0.05, statistically significant difference; **P < 0.001, highly statistically significant difference.

**FIGURE 3:**
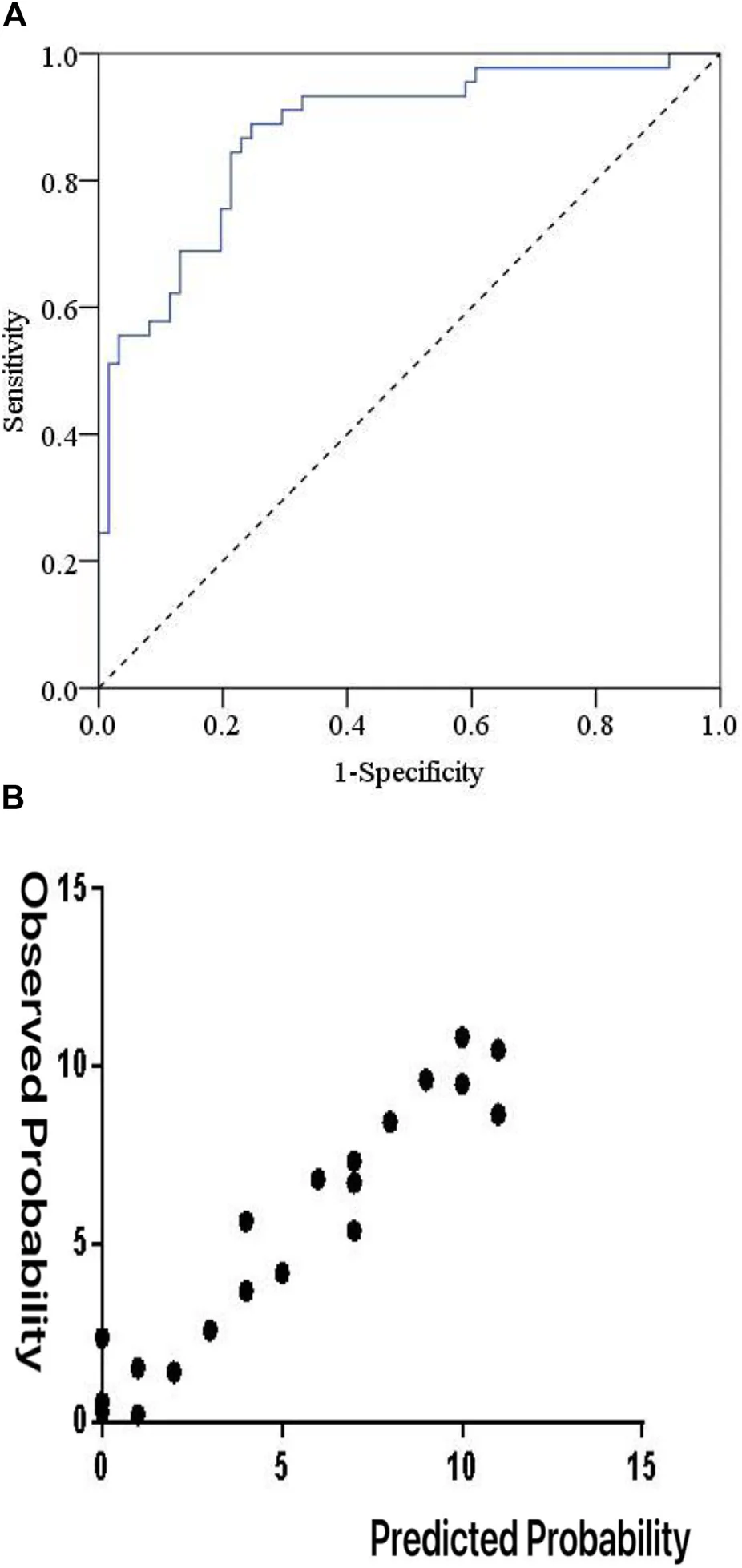
**(A)** The ROC curve of the high-frequency ultrasound model for the diagnosis of Duchenne muscular Dystrophylar dystrophy (DMD). **(B)** Scatter plot of the high-frequency Hosmer-Lemeshow (HL) Test.

### Diagnostic accuracy of the SWE model

We used univariate and multivariate logistic regression analysis to build the SWE model in diagnosing DMD. Univariate logistic regression analysis revealed that the shear wave velocities of the right biceps brachii (RBB-SWV), right vastus lateralis (RVL-SWV), left vastus intermedius (LVI-SWV), and left vastus medialis (LVM-SWV) muscles were potential factors in diagnosing DMD. Multivariate logistic regression analysis showed that RBB-SWV (odds ratio [OR] = 0.170, 95% confidence interval [CI]: 0.063–0.460, P < 0.001), RVL-SWV (OR = 3.836, 95% CI: 1.623–9.070, *P* = 0.002), and LVI-SWV (OR = 2.844, 95% CI: 1.002–8.075, *P* = 0.050) were independent predictive factors for the diagnosis of DMD (Table 5).

**TABLE 5:** Single and multiple factor analysis of elasticity variable in DMD.

Muscle type	Single factor logistic regression analysis			Multivariate logistic regression analysis		
​	OR	95 % CI	*P*	OR	95 % CI	*P*
Right biceps brachii	0.150	0.060–0.374	<0.001	0.170	0.063–0.460	<0.001**
Right lateral thigh muscle	3.778	1.788–7.983	<0.001	3.836	1.623–9.070	0.002*
Left intermuscular fascia of the left femur	3.365	1.469–7.705	0.004	2.844	1.002–8.075	0.05*
Left medial gastrocnemius muscle mean	0.202	0.074–0.548	0.002	0.322	0.102–1.011	0.052

*P < 0.05, statistically significant difference; **P < 0.001, highly statistically significant difference.

Based on the Youden index, the optimal cut-off values for RVL-SWV, RVI-SWV, and LVI-SWV were determined to be 3.083 m/s, 2.943 m/s, and 2.617 m/s, respectively. For the logistic regression model, these continuous variables were dichotomized. A score of “1″was assigned if the value indicated a higher risk for DMD: RVL-SWV ≤3.083 m/s, RVI-SWV >2.943 m/s, or LVI-SWV >2.617 m/s. Conversely, a score of “0″was assigned for values suggesting a lower risk (Table 6 and 7).

**TABLE 6:** Logistic regression cut-off values of each variable in the elastography model.

Muscle type	Sensibility %	Specificity%	PPV %	NPV %	Truncated value	AUC	95 % CI	P
Right biceps brachii	69.8	78.7	74.0	75.0	3.083	0.777	0.690–0.850	<0.001**
Right lateral femoral muscle	62.3	73.8	67.3	69.2	2.943	0.708	0.615–0.789	<0.001**
Left musculus medialis femoris	58.5	70.5	63.3	66.2	2.617	0.650	0.556–0.737	0.0036*

*P < 0.05, statistically significant difference; **P < 0.001, highly statistically significant difference

**TABLE 7:** Logistic regression scoring table of each variable in the elastography model.

Muscle type	Truncated value, Csmean	Evaluation
Right biceps brachii	>3.083	0
	≤3.083	1
Right lateral femoral muscle	≤2.943	0
	>2.943	1
Left musculus medialis femoris	≤2.617	0
	>2.617	1

The area under the ROC curve of the elastic ultrasound model for diagnosing DMD is 0.846 (95% confidence interval (CI): 0.763–0.909, *P* < 0.001), with a sensitivity of 0.846 and specificity of 0.698 (Figure 4).

**FIGURE 4:**
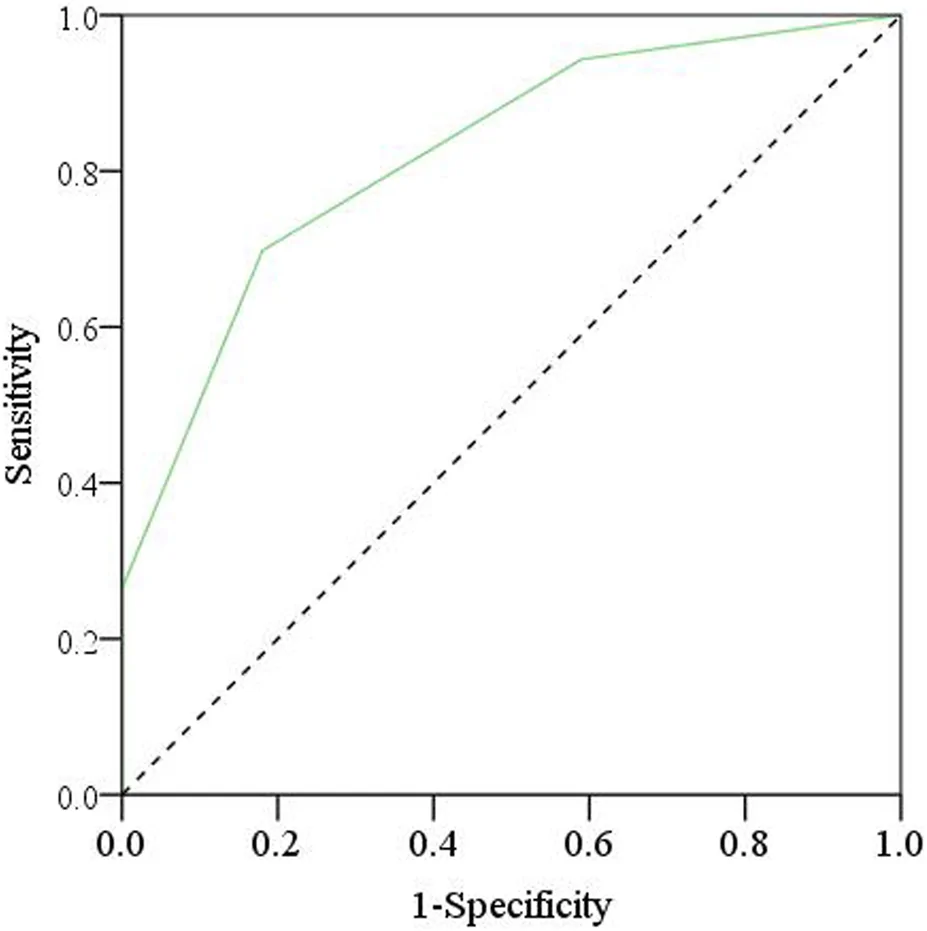
The ROC Curve of shear-wave elastography (SWE) Model for the Diagnosis of DMD, AUC = 0.846, (95% CI 0.763–0.909), P < 0.001.

### Comparison and combination of high-frequency ultrasound model and SWE model

The area under the curve (AUC) of the HFUS model is 0.875 (95% CI: 0.763–0.909), and the AUC of the SWE model is 0.846 (95% CI: 0.797–0.931). We then compared and found that there was no significant statistical difference between the two models (0.875 vs. 0.846, *P* = 0.494).

We then combined the two models for the diagnosis of DMD, the AUC of the combined model increases to 0.947 (95% CI: 0.885–0.981), which is better than that of the HFUS model alone (0.947 vs. 0.875, *P* < 0.001) or the SWE model alone (0.947 vs. 0.846, *P* < 0.001). The sensitivity of the combined model is 0.822 and the specificity is 0.951 (Figure 5).

**FIGURE 5:**
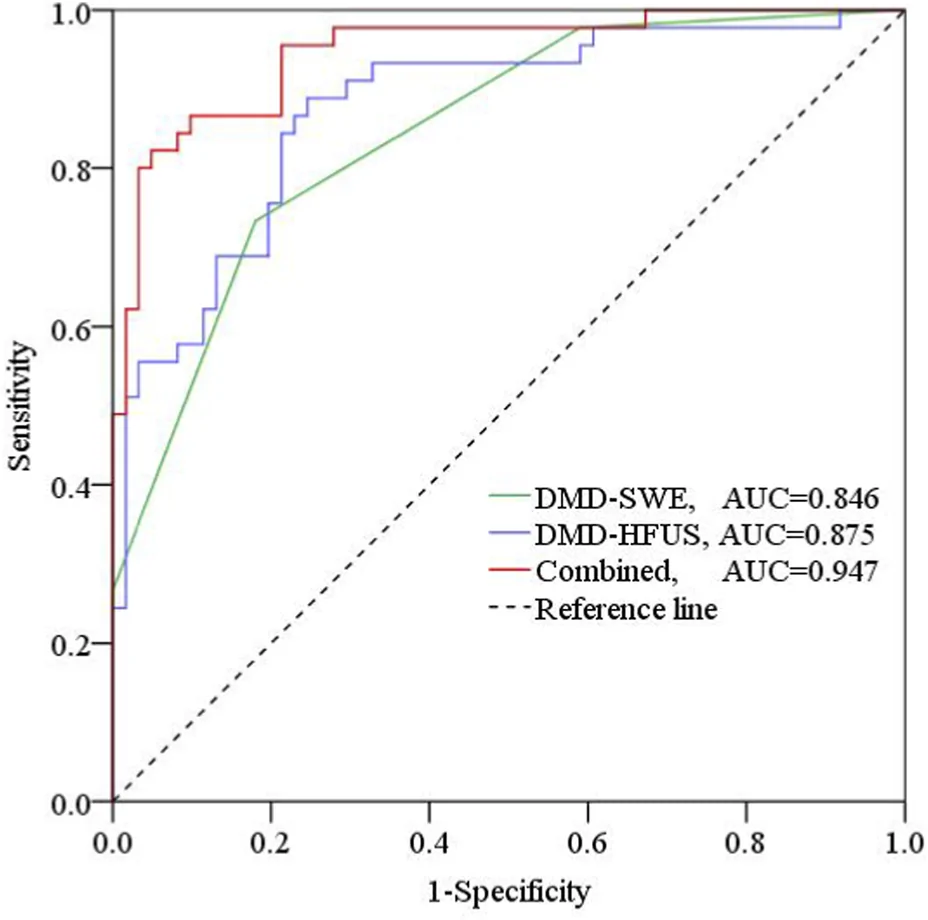
The ROC curves of the high-frequency model, the shear-wave elastography (SWE) model, and their combined model for the diagnosis of Duchenne muscular dystrophy (DMD).

We then combined the two models for the diagnosis of DMD, and the AUC of the combined model increased to 0.947 (95% CI: 0.885–0.981), which is better than that of the HFUS model alone (0.947 vs. 0.875, *P* < 0.001) or the SWE model alone (0.947 vs. 0.846, *P* < 0.001). The sensitivity of the combined model is 0.822 and the specificity is 0.951 (Figure 5). For the combined HFUS-SWE model, the positive predictive value (PPV) was 94.3% and the negative predictive value (NPV) was 84.8% at the prevalence observed in our study cohort.

The decision curve analysis (DCA) showed that the net benefit of the combined model for diagnosing DMD is higher than that of either the HFUS model or the SWE model at any threshold probability. There is no significant difference in the net benefit between the HFUS model and the SWE model (Figure 6).

**FIGURE 6:**
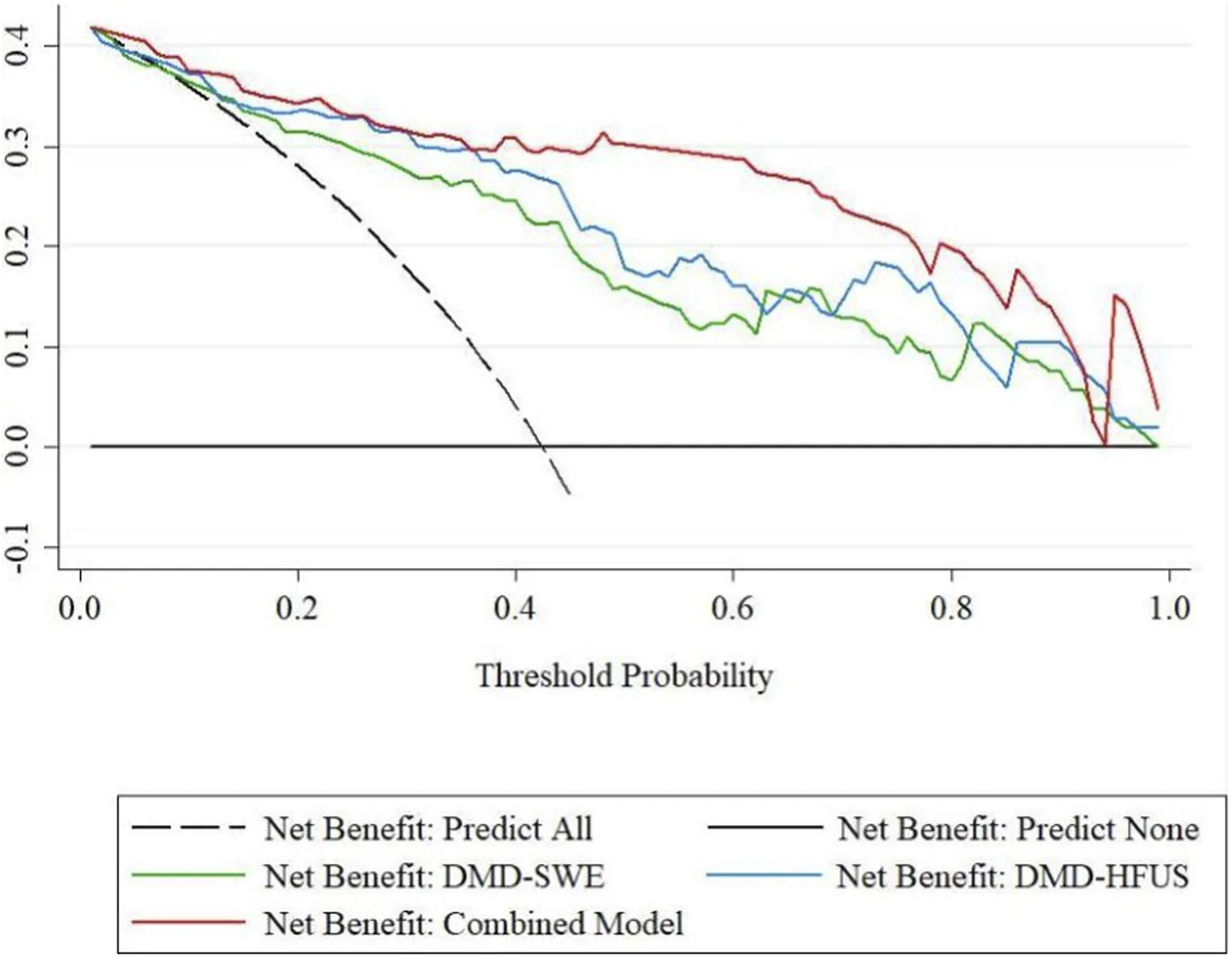
The DCA curves of the high-frequency model, the shear-wave elastography (SWE) model, and their combined model for the Diagnosis of Duchenne muscular dystrophy (DMD).

## Discussion

A statistically significant difference in height was observed between the DMD and control groups. This finding is consistent with the known growth retardation commonly seen in boys with DMD, which may be attributed to a combination of factors including chronic disease, malnutrition, delayed puberty, and the use of corticosteroids. While height could theoretically influence muscle biomechanical properties (e.g.,), muscle length, and resting tension), the magnitude of the observed SWE changes in DMD is substantially larger than what would be expected from height differences alone. Furthermore, our HFUS and SWE models were based on standardized measurement sites and relative ratios, which minimize the confounding effect of absolute body size.

This study presents a novel diagnostic framework for DMD by developing and validating models based on HFUS and SWE. Our analysis of 3,420 parameters from 1,140 muscle sites in 53 pediatric DMD patients and 61 healthy controls yielded three key findings. First, the HFUS model, constructed via principal component analysis, demonstrated high diagnostic accuracy with an AUC of 0.875. Second, the SWE model identified independent predictors—specifically, the shear wave velocities of the right biceps brachii, right vastus lateralis, and left vastus intermedius—with an AUC of 0.846. Crucially, no significant difference was found between the diagnostic efficacy of the two individual models (*P* = 0.494). The most significant finding was that the combined HFUS-SWE model achieved a superior AUC of 0.947, significantly outperforming either model alone (*P* < 0.001), indicating a powerful synergistic effect.

Our findings on HFUS align with and quantitatively extend the existing understanding of DMD pathophysiology. The observed progressive increase in muscle echogenicity, particularly in the lower limbs, is a well-documented sonographic hallmark of DMD, corresponding histologically to fibrofatty infiltration replacing musclee fibers (Boon et al., 2021; Ferreira et al., 2016; Yu et al., 2022). We further quantified structural degeneration through measurements of reduced muscle thickness and cross-sectional area, consistent with studies by Ferreira et al. (2016) on masticatory muscles and others on limb girdle muscles (Lin et al., 2023; Lee et al., 2023; Kara et al., 2021). This widespread atrophy results from recurrent cycles of muscle fiber necrosis and failed regeneration. The high inter- and intra-observer reliability (ICC >0.9) we observed for HFUS parameters corroborates previous reports and underscores its robustness for longitudinal monitoring.

The inter-observer reliability for both HFUS and SWE measurements was excellent. The intraclass correlation coefficients (ICC) for muscle thickness, cross-sectional area, and shear wave velocity measurements ranged from 0.92 to 0.98, indicating high consistency between the two independent readers.

The application of SWE in DMD is an emerging field. Our finding of significantly reduced SWV in DMD patients reflects a decrease in muscle stiffness, likely due to the replacement of stiff, contractile muscle tissue with softer adipose and connective tissue. This contrasts with studies on spastic conditions like cerebral palsy, where muscle stiffness is increased (Tagliafico et al., 2022). The more pronounced softening in the lower extremities, and this finding is consistent with MRI studies that demonstrate earlier and more severe fat infiltration in these specific muscle groups (Pichiecchio et al., 2018; Abdulhady et al., 2022). The heterogeneity in stiffness between left and right homologous muscles, also noted by other researchers, may reflect the asymmetric and patchy nature of the disease process (Goo et al., 2024). Our establishment of specific cut-off values (e.g., 2.617 m/s for the vastus intermedius) provides a preliminary quantitative basis for SWE diagnosis.

The superior performance of the combined HFUS-SWE model is the cornerstone of our study. It strongly suggests that HFUS and SWE capture complementary pathophysiological information: HFUS primarily reflects macroscopic morphological changes (atrophy and architecture disruption), while SWE assesses microstructural biomechanical properties (tissue stiffness). This synergy enables the combined model to overcome the limitations of each standalone technique, achieving both high sensitivity (82.2%) and exceptional specificity (95.1%). In our cohort, the combined model yielded a positive predictive value (PPV) of 94.3% and a negative predictive value (NPV) of 84.8%, indicating strong clinical utility for risk stratification.

While genetic testing remains the diagnostic gold standard (and is now available via non-invasive saliva samples), it is resource-intensive and may not be readily accessible in all primary care settings. Therefore, our combined HFUS-SWE model is best positioned as a non-invasive, point-of-care screening and triage tool. It can help prioritize patients with a high pretest probability for confirmatory genetic testing, while its high NPV can aid in low-risk cases, thereby reducing unnecessary referrals and parental anxiety. It is crucial to interpret the predictive values (PPV and NPV) reported in our study with caution, as these are highly dependent on disease prevalence. Our study cohort had an artificially high prevalence of 46.5%, which inflates the PPV and NPV compared to a general screening scenario where the prevalence is approximately one in 3,500–5,000. Therefore, in a real-world primary care setting, the NPV would be significantly higher (close to 100%) and the PPV would be substantially lower. The true strength of our combined model lies not in ruling out DMD confidently, but in its high negative likelihood ratio and positive likelihood ratio, which can effectively shift the pre-test probability and help clinicians prioritize which children should be referred for the costly and resource-intensive confirmatory genetic testing. This approach is particularly valuable for risk stratification in primary care settings. This is a distinct advantage over serum CK testing, which, although sensitive, lacks specificity (Birnkrant et al., 2018b; Kim et al., 2017).

Our findings suggest a potential clinical workflow: initial screening with serum CK and basic HFUS of lower limb muscles, followed by a comprehensive HFUS-SWE examination for children with suspicious findings. A positive result on the combined model could then prioritize patients for genetic testing. This approach could be particularly valuable in primary care or resource-limited settings where access to genetic testing is delayed.

Limitations of our study should be acknowledged. Its single-center, retrospective design and relatively small sample size may limit the generalizability of our findings. While we focused on muscle-specific parameters, the potential confounding effect of subcutaneous fat was considered but not quantified due to its compressibility. Future prospective, multi-center studies with larger cohorts are warranted to validate and refine our models.

## Conclusion

This study demonstrates that diagnostic models based on HFUS and SWE are effective in diagnosing DMD in children. The combined HFUS-SWE model exhibits outstanding diagnostic performance, highlighting the complementary value of assessing both muscle morphology and biomechanical properties. This integrated imaging approach holds great promise as a valuable non-invasive tool for the risk stratification and long-term monitoring of DMD, potentially improving clinical management and patient outcomes.

## Data Availability

The original contributions presented in the study are included in the article/supplementary material, further inquiries can be directed to the corresponding authors.
